# Supplementary material for: Solid-State Formation of a Potential Melphalan Delivery Nanosystem Based on β-Cyclodextrin and Silver Nanoparticles
Source: Int J Mol Sci. 2023 Feb 16;24(4):3990. doi: 10.3390/ijms24043990 (PMC9964812; doi:10.3390/ijms24043990)
Supplement: Supplementary file 1 [file ijms-24-03990-s001.zip › ijms-2182251-supplementary.pdf]

International Journal of *Molecular Sciences*

MDPI

Supporting Information

Solid-state formation of a potential Melphalan delivery nanosystem based on β-cyclodextrin and silver nanoparticles.

Rodrigo Sierpe [1,2,3], Orlando Donoso-González [1,2,4,5], Erika Lang [1], Michael Noyong [5], Ulrich Simon [5], Marcelo J. Kogan [2,4,*] and Nicolás Yutronic [1,*]

[1] Departamento de Química, Facultad de Ciencias, Universidad de Chile, Las Palmeras #3425, Ñuñoa, Santiago, Chile
[2] Departamento de Química Farmacológica y Toxicológica, Facultad de Ciencias Químicas y Farmacéuticas, Universidad de Chile, Sergio Livingstone #1007, Independencia, Santiago, Chile
[3] Departamento de Química, Facultad de Ciencias Naturales, Matemática y del Medio Ambiente, Universidad Tecnológica Metropolitana (UTEM), Las Palmeras 3360, Ñuñoa, 7800003 Santiago, Chile
[4] Advanced Center for Chronic Diseases (ACCDiS), Sergio Livingstone #1007, Independencia, Santiago, Chile
[5] RWTH Aachen University, Institute of Inorganic Chemistry, Landoltweg 1a, D-52074 Aachen, Germany
* Correspondence: nyutroni@uchile.cl (N.Y.), mkogan@ciq.uchile.cl (M.K.)

Citation: To be added by editorial staff during production.

Academic Editor: Firstname Lastname

Received: date
Revised: date
Accepted: date
Published: date

Copyright: © 2023 by the authors. Submitted for possible open access publication under the terms and conditions of the Creative Commons Attribution (CC BY) license (https://creativecommons.org/licenses/by/4.0/).

S1. Stoichiometric ratio of the inclusion complex

The stoichiometric ratios were calculated in the ^{1}H-NMR spectra by comparing the integrals of the Mel protons with the integrals of the βCD protons from the βCD-Mel systems. First, the integrals of the Mel signals (protons H'2'/6' and H'3/5') were analyzed using the H1 signal of βCD as reference, which integrated for 7 as it is possible to observe in figure S1. The stoichiometric ratios calculated are summarized in Table S1. The calculated stoichiometric ratio was 1:1.

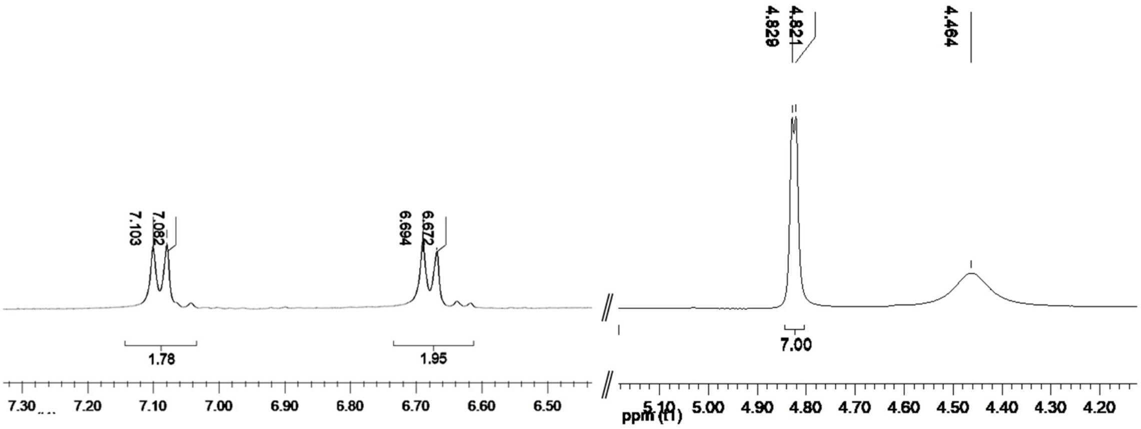

Figure S1. Signal integration of ^{1}H-NMR spectra of βCD-Mel in DMSO-d_6.

Table S1. Values of the integrated Mel and βCD proton signals in the ^{1}H-NMR spectra of the βCD-Mel system, with the integrated H1 proton signals of βCD as a reference.

Proton signal	Reference	Integral	Counts	Ratios
H1	7	7	1	1
H'2'/6'	2	1.78	0.89	1
H'3/5'	2	1.95	0.98	1

S2. Determination of association constants of inclusion complex

To perform the quantification of Mel, a calibration curve was made with stocks of aqueous solutions of known concentrations according to the absorbance maxima at 301 nm, to obtain the value of ε of Beer-Lambert law. Figure S2 shows the UV-vis spectra of known concentrations of aqueous Mel solutions.

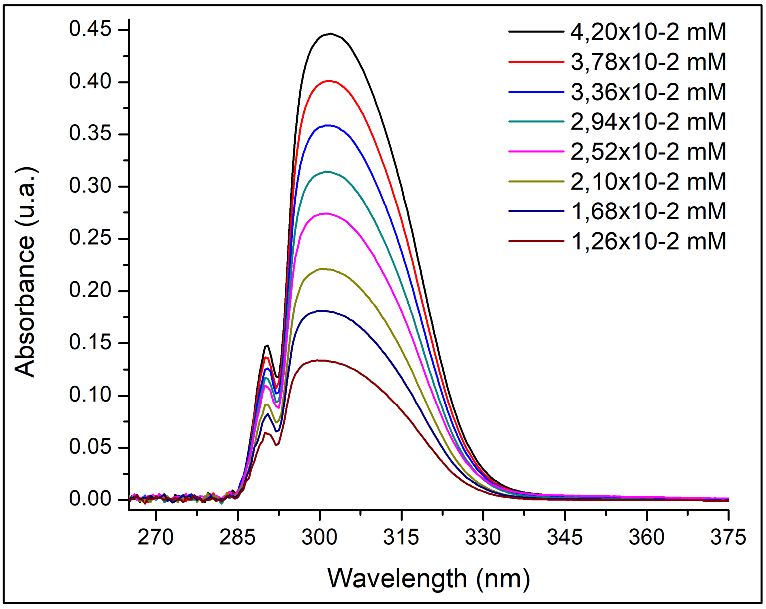

Figure S2. UV-vis spectra of different concentrations of aqueous Mel solutions.

The absorbance maxima of the UV-vis spectra at 301 nm and the concentration of the Mel solution were attached in Table S2.

Table S2. Data on the concentrations of the drug and its absorbance maxima at 301 nm, respectively.

[Mel] (mmol/L)	Absorbance (a.u.)
0.0420	0.4452
0.0378	0.4010
0.0336	0.3585
0.0294	0.3143
0.0252	0.2738
0.0210	0.2212
0.0168	0.1811
0.0126	0.1335

By plotting the Mel concentration versus the maximum absorbance at 301 nm, the line shown in Figure S3 was obtained. The linear range with an R-square of 0.99993 indicates a correct fit. The value of ε was 10.6534 ± 0.0324 $mM^{-1}cm^{-1}$.

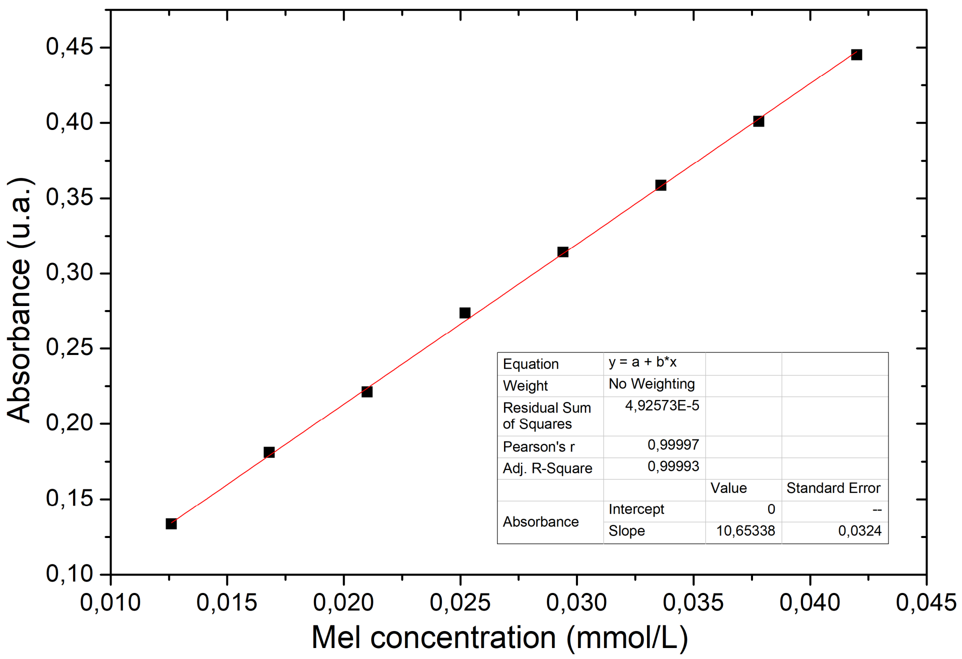

Figure S3. Linear plots of the Mel absorbance maxima at 301 nm vs. concentrations.

For the determination of the *Ka* values, a stock solution was prepared with 200 mg of βCD and water in a 25 mL measuring flask. Volumes of 0 to 2 mL of the stock were taken and diluted with water to produce a total volume of 2 mL with a fixed amount of the drug, 5.0 mg of Mel was added. All the data obtained are presented in Table S3. By applying the extinction coefficient value ε to equation 1, it was possible to determine Mel concentrations present in the different assays using the Higucchi-Connors model.

Table S3. Values of the tests made to calculate the *Ka* and CE of the βCD-Mel system in water.

[βCD] (mmol/L)	Absorbance (a.u.)	[Mel] (mmol/L)
0	0.5871	0.05511
0.0440	0.5988	0.05621
0.0881	0.6271	0.05886
0.1760	0.6691	0.06281
0.2200	0.6712	0.06300
0.2643	0.6772	0.06357
0.3524	0.7305	0.06857
0.4400	0.7359	0.06908

The linear relationship obtained from a plot of the solubilized Mel concentration versus the added βCD concentration is shown in Figure S4. The value of the slope was 0.0334 (± 0.0026). Using equation 1, the association constant *Ka* was calculated, resulting in a value of 625 M^{-1}. Finally, using equation 2, the value of complexation efficiency (CE) was calculated, resulting in a value of 0.035 for the βCD-Mel system.

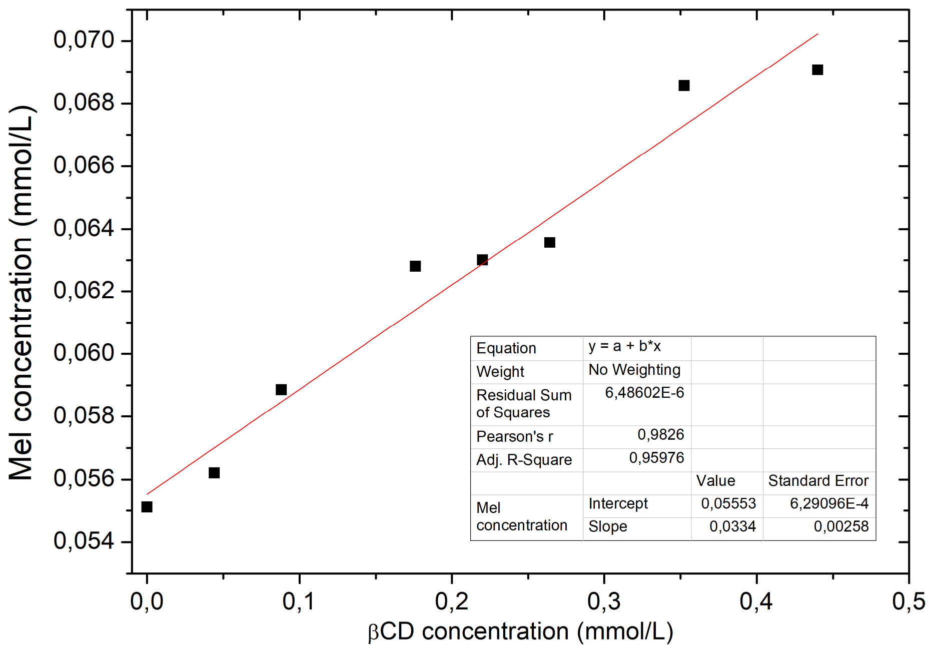

Figure S4. Graph of the concentration of solubilized Mel versus the concentration of added βCD and the linear fit.

S3. Full ROESY spectra of the inclusion complex

Figure S5 shows the complete ROESY spectrum of the βCD-Mel system, from which the zooms made for Figure 5a and 5b were then obtained.

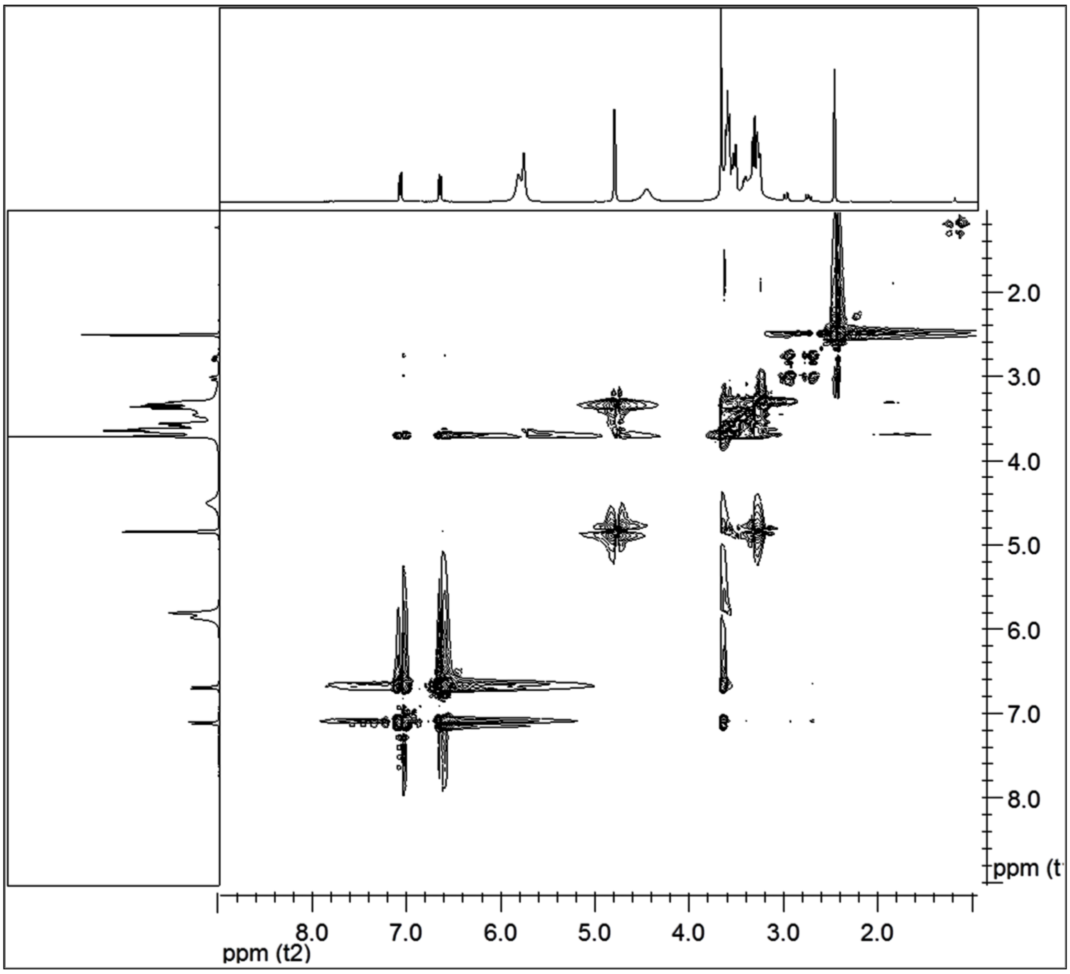

Figure S5. Full ROESY spectra of βCD-Mel complex in DMSO-d_6.

S4. UV-vis spectra of melphalan in solid state

Figure S6 shows UV-vis spectra of Mel in solid state.

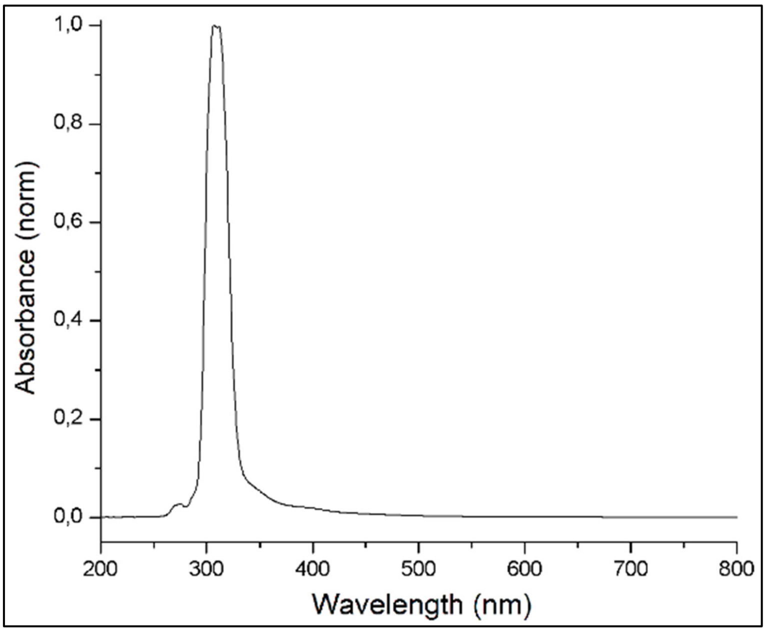

Figure S6. UV-vis spectra of Mel in solid state.

S5. More FE-SEM and EDX of inclusion complex with silver nanoparticles

Figure S7 shows FE-SEM micrographs of βCD-Mel crystals covered with AgNPs.

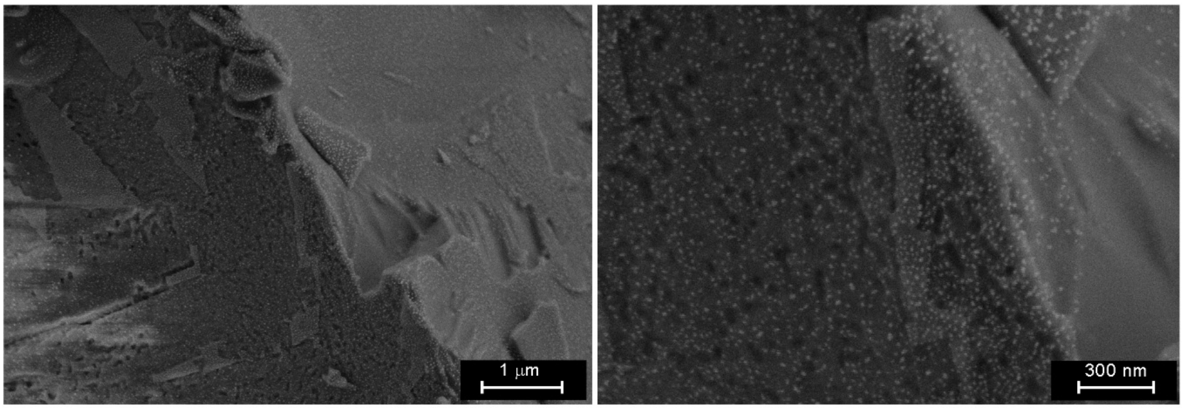

Figure S7. FE-SEM micrograph of βCD-Mel with AgNPs, the time of exposure in sputtering was 32 s.

The data collected from the different EDX spectra were summarized in Table S4.

Table S4. Average percentage of each element in sections from FE-SEM images.

Element	Average weight (%)
C K	53.2±6.4
O K	46.3±6.5
Cl K	0.19±0.09
Ag L	0.29±0.05
**Total**	**100,000**

S6. SEM of β-cyclodextrin, melphalan, and inclusion complex

Figure S8 shows SEM micrographs of (A) βCD, (B) Mel and (C) βCD-Mel crystals, which were previously covered with silver atoms to improve their conductivity.

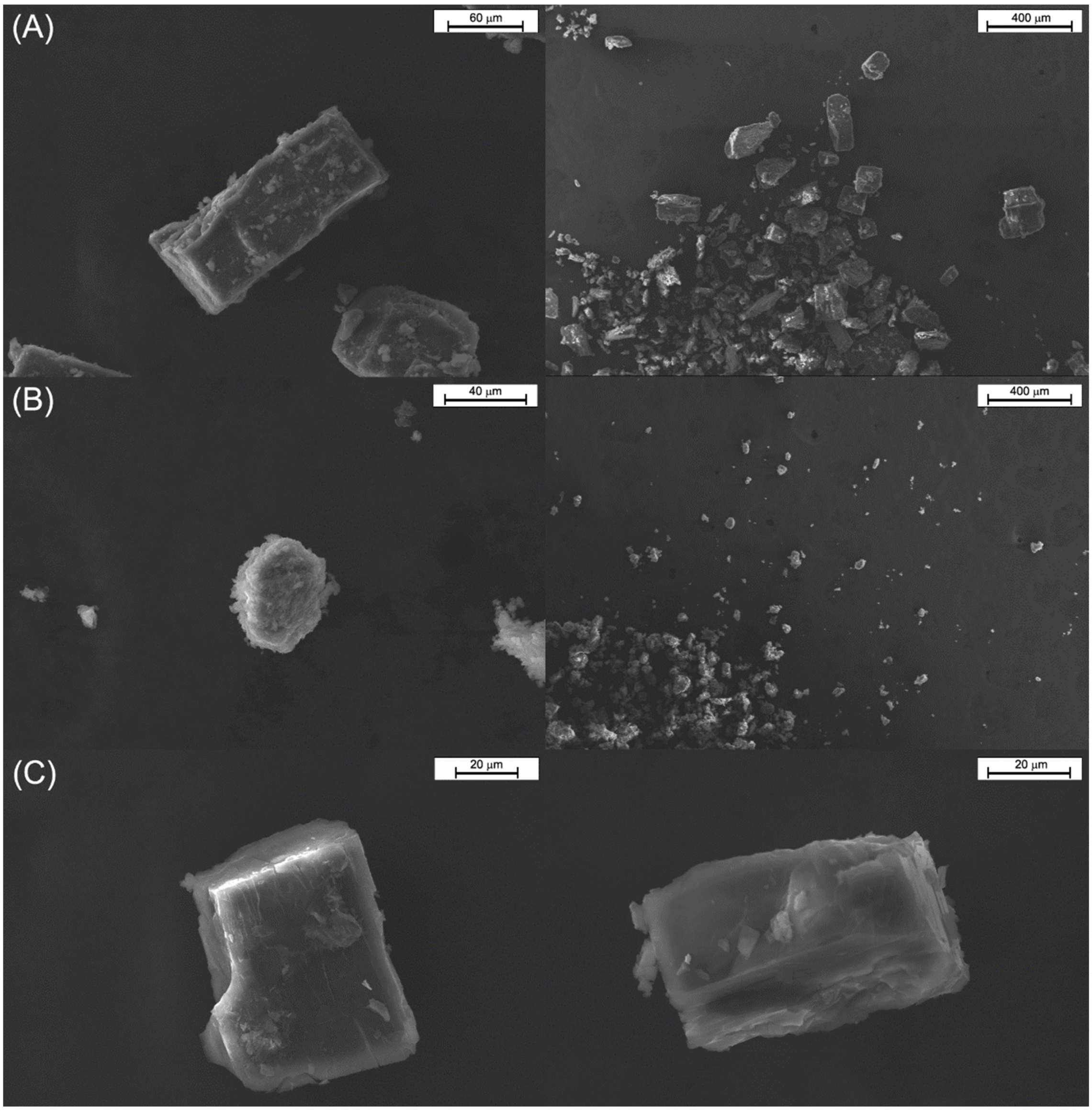

Figure S8. SEM micrographs of (A) βCD, (B) Mel and (C) βCD-Mel crystals.

S7. Parallel artificial membrane permeability assay methods and analysis

PAMPA was performed in triplicate (n = 3), with each n representing an average of three assays performed on the same plate. Table S5 shows the absorbance values, calculated concentrations and effective permeabilities of the evaluated systems: Mel, βCD-Mel, βCD-Mel-AgNPs.

Table S5. Absorbances, concentrations and effective permeabilities obtained via PAMPA.

System	Absorbance (a.u.)	Concentration (μmol/mL)	Effective permeability (cm/s)
Mel	0.07	0.0070	$1.044x10^{-7}$
	0.159	0.0159	$2.412x10^{-7}$
	0.124	0.0124	$1.869x10^{-7}$
βCD-Mel	0.666	0.0665	$1.241x10^{-5}$
	0.592	0.0591	$7.508x10^{-6}$
	0.536	0.0535	$5.807x10^{-6}$
βCD-Mel-AgNPs	0.151	0.0151	$9.660x10^{-7}$
	0.194	0.0194	$1.291x10^{-6}$
	0.116	0.0116	$7.202x10^{-7}$

Disclaimer/Publisher's Note: The statements, opinions and data contained in all publications are solely those of the individual author(s) and contributor(s) and not of MDPI and/or the editor(s). MDPI and/or the editor(s) disclaim responsibility for any injury to people or property resulting from any ideas, methods, instructions or products referred to in the content.
